# Chromatographic analysis of Polygalae Radix by online hyphenating pressurized liquid extraction

**DOI:** 10.1038/srep27303

**Published:** 2016-06-07

**Authors:** Yuelin Song, Qingqing Song, Jun Li, Shepo Shi, Liping Guo, Yunfang Zhao, Yong Jiang, Pengfei Tu

**Affiliations:** 1 Modern Research Center for Traditional Chinese Medicine, Beijing University of Chinese Medicine, Beijing 100029, China; 2 School of Chinese Materia Medica, Beijing University of Chinese Medicine, Beijing 100102, China; 3 Thermo-Fisher Scientific Corporation, Shanghai 201205, China; 4 State Key Laboratory of Natural and Biomimetic Drugs, School of Pharmaceutical Sciences, Peking University, Beijing 100191, China

## Abstract

Practicing “green analytical chemistry” is of great importance when profiling the chemical composition of complex matrices. Herein, a novel hybrid analytical platform was developed for direct chemical analysis of complex matrices by online hyphenating pressurized warm water extraction followed by turbulent flow chromatography coupled with high performance liquid chromatography-tandem mass spectrometry (PWWE-TFC-LC-MS/MS). Two parallel hollow guard columns acted as extraction vessels connected to a long narrow polyether ether ketone tube, while warm water served as extraction solvent and was delivered at a flow rate of 2.5 mL/min to generate considerable back pressure at either vessel. A column oven heated both the solvent and crude materials. A TFC column, which is advantageous for the comprehensive trapping of small molecular substances from fluids under turbulent flow conditions, was employed to transfer analytes from the PWWE module to LC-MS/MS. Two electronic valves alternated each vessel between extraction and elution phases. As a proof-of-concept, a famous herbal medicine for the treatment of neurodegenerative disorders, namely Polygalae Radix, was selected for the qualitative and quantitative analyses. The results suggest that the hybrid platform is advantageous in terms of decreasing time, material, and solvent consumption and in its automation, versatility, and environmental friendliness.

Natural products have been widely preferred as an ideal source for the discovery of drug leads/new chemical entities (NCEs)^1^; however, the complex chemical composition of herbs, microbes, and marine organisms make the extraction process as well as the chemical analysis challenging. Tedious pre-process procedures and large quantities of organic solvents are usually involved, which leads to negative impacts on the environment and human health. Hence, green, efficient, and automated extraction processes as well as direct analysis methods for natural products in biomass, which coincide with the “green analytical chemistry” concept^2^, have become increasingly important in the pharmaceutical and biochemical industries.

Conventional extraction techniques, such as Soxhlet extraction, sonication, and solid–liquid extraction^3^, suffer from labor intensive procedures, and the large amounts of materials and organic solvents involved are often costly to purchase and dispose of, in addition to their negative impacts on the environment or human health^4^,^5^. Moreover, the extracts obtained from traditional approaches often require subsequent laborious processing procedures, such as concentration and reconstitution, prior to analysis. Pressurized liquid extraction (PLE), especially employing water as the extraction solvent, has been demonstrated to be an emerging greener technology^6^,^7^,^8^. The water polarity dramatically decreases with increasing temperature because of the hydrogen bond dissolution and the use of elevated temperature and pressure, and the water polarity reaches a level comparable with organic solvent-water mixtures. Hence, warm water exhibits the potential to dissolve a greater amount of semi-polar compounds^9^,^10^. In addition, the lower viscosity and surface tension of the heated water enhance the penetration capability of water into the sample matrix and mass transfer rates of the compounds from the plant tissue matrix, which improves the extraction efficiency^11^. Therefore, PLE with warm water could not only improve the extraction yield but also decrease the extraction time and the material and solvent consumption^3^. However, expensive, specialized, and sophisticated apparatuses are usually required for providing the pressure and for heating, and it is, therefore, a difficult task to achieve compatibility between the PLE equipment and the analytical platforms used. Consequently, to achieve the “green analytical chemistry” goal, especially the principle of direct analysis^12^, more researches are required for the online connection of PLE and analytical platforms^13^.

Thus far, several preliminary attempts have been devoted to the online integration of PLE and solid phase extraction (SPE)^14^,^15^, the latter of which is a widely favored technique for online sample preparation for analytical platforms. However, neither practical instrumentation or further integration of PLE, SPE, and an analytical platform have been accomplished. A feasible solution is to modify the PLE domain to overcome the incompatibility. It is well known that a high pressure can be generated by turbulent flows in a narrow tube; thus, a long narrow tube can be used to provide the desired back pressure for crude materials that are contained in an appropriate vessel. Herein, a novel and facile pressurized warm water extraction (PWWE) module was designed (Fig. 1), in which a hollow guard column (3.0 mm I.D. ×4.0 mm) acted as an extraction vessel that was connected to the rest of the system via a long polyether ether ketone (PEEK) tube (0.13 mm I.D. ×1000 mm), and a solvent delivery unit was used to deliver the extraction solvent at a flow rate of 2.5 mL/min to generate a high back pressure (approximately 13.0 MPa). The column oven was maintained at 75 °C and warmed both the solvent and crude materials to achieve accelerated solvent extraction. Because pressurized warm water, which is readily available, non-toxic and can be recycled or disposed of with minimal environmental problems, is capable of extracting semi-polar components, it is a green solvent and can replace organic solvents, such as methanol and acetonitrile (ACN). After the extraction, because the PWWE module was composed of several LC units, it is convenient to hyphenate it with high performance liquid chromatography-tandem mass spectrometry (LC-MS/MS), which is one of the workhorses for analyte detection, by introducing an online SPE column. A high solvent flow-rate is beneficial to PLE for extracting crude materials, however, it creates challenges for the extraction efficiency of the SPE column. Fortunately, this requirement fits well with the principle of turbulent flow chromatography (TFC), which is a special SPE technique that is useful for the comprehensive extraction of small molecular weight components from fluids under turbulent flow conditions inside a TFC column. Therefore, an analytical platform was developed to accomplish online PWWE-TFC-LC-MS/MS (Fig. 2). Two electronic 2-channel/6-port valves were used to switch two parallel extraction vessels between the extraction and elution phases. LC-MS/MS was used to receive, separate, and detect the analytes that were transferred from the TFC column in the elution phase. As a proof of concept, Polygalae Radix, the dried roots of *Polygala tenuifolia* Willd, which is one of the most widely used traditional Chinese medicines and plays an important role for improving the ecological environments of the Loess Plateau in China, was employed as a case study to validate the applicability of online PWWE-TFC-LC-MS/MS. Moreover, this famous herbal medicine has also been demonstrated as a promising candidate for the treatment of neurodegenerative disorders, *e.g.*, Alzheimer’s disease (AD)^16^,^17^.

## Results

### Comparisons of the extraction efficiencies among PWWE, reflux, and sonication strategies

**Figure 1:**
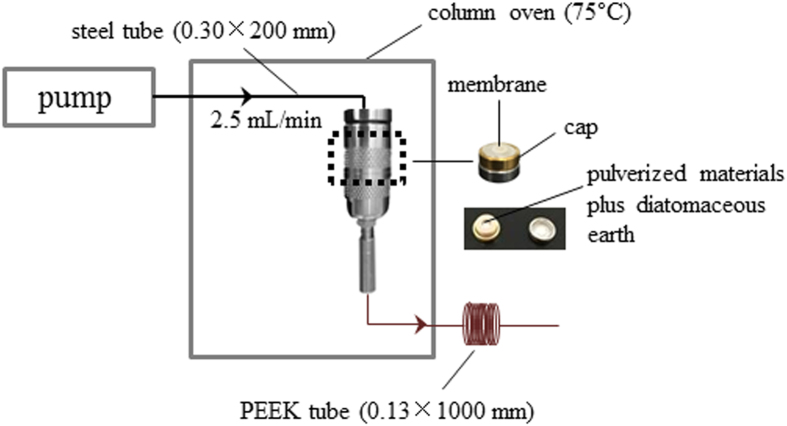
Schematic diagram of the online PWWE module. Detailed descriptions are included in the section “Configuration of PWWE module and assessment of the extraction efficiency.”

PLE usually requires fit-for-purpose equipment, *e.g.*, the Dionex ASE system (Sunnyvale, CA, USA), and an inert gas, such as nitrogen, is always utilized to generate the pressure required to accelerate the extraction process^18^,^19^, which is a significant barrier for the online coupling of PLE with LC-based analytical platforms. Because significant pressure can result from high flow rates in a narrow tube, a long narrow tube can be implemented to achieve the desired back pressure for crude materials that are stored in an appropriate vessel. Therefore, a PWWE module (Fig. 1) was configured by employing several conventional LC units, including a pump, a column oven, a hollow guard column with its corresponding holder, a steel tube, and a long PEEK tube to link the PWWE module to the LC-MS/MS. Because pressurized warm water, which is readily available, non-toxic, and can be recycled or disposed with minimal environmental problems, is capable of extracting semi-polar components from solid matrices, it acted as a green solvent instead of organic solvents, such as methanol and ACN.

The extracts obtained by PWWE, sonication with 70% aqueous methanol, sonication with pure water, and reflux with 70% aqueous methanol were compared using LC-IT-TOF-MS. The base peak chromatograms (BPCs) are shown in Fig. S1A–D (Supplemental information). A significant similarity was observed in the overall profiles of the four subfigures, which suggests that all the extracts shared similar chemical compositions regarding qualitative and quantitative characteristics; however, some differences were also observed. Higher responses for the peaks that eluted before 10 min are shown in Fig. S1A compared with Fig. S1B,D, whereas comparable responses are observed for the peaks that eluted after 10 min among Fig. S1A,B and D. Therefore, the PWWE module showed a slightly higher extraction efficiency for those hydrophilic substances than either sonication with 70% aqueous methanol or reflux with 70% aqueous methanol, without sacrificing the extraction capacity for low-polarity compounds. Moreover, comparable responses are shown for the peaks that eluted before 10 min in Fig. S1A,C, whereas Fig. S1A shows higher responses for the components that eluted after 10 min than those in Fig. S1C, which suggests that the PWWE module provided a greater extraction efficiency than sonication with pure water, in particular for the less polar compounds. Thus, PWWE was demonstrated as a feasible approach to achieve the green and comprehensive extraction of components in crude materials, using a water-based extraction time of only three minutes.

### Configuration of the online PWWE-TFC-LC-MS/MS system

**Figure 2:**
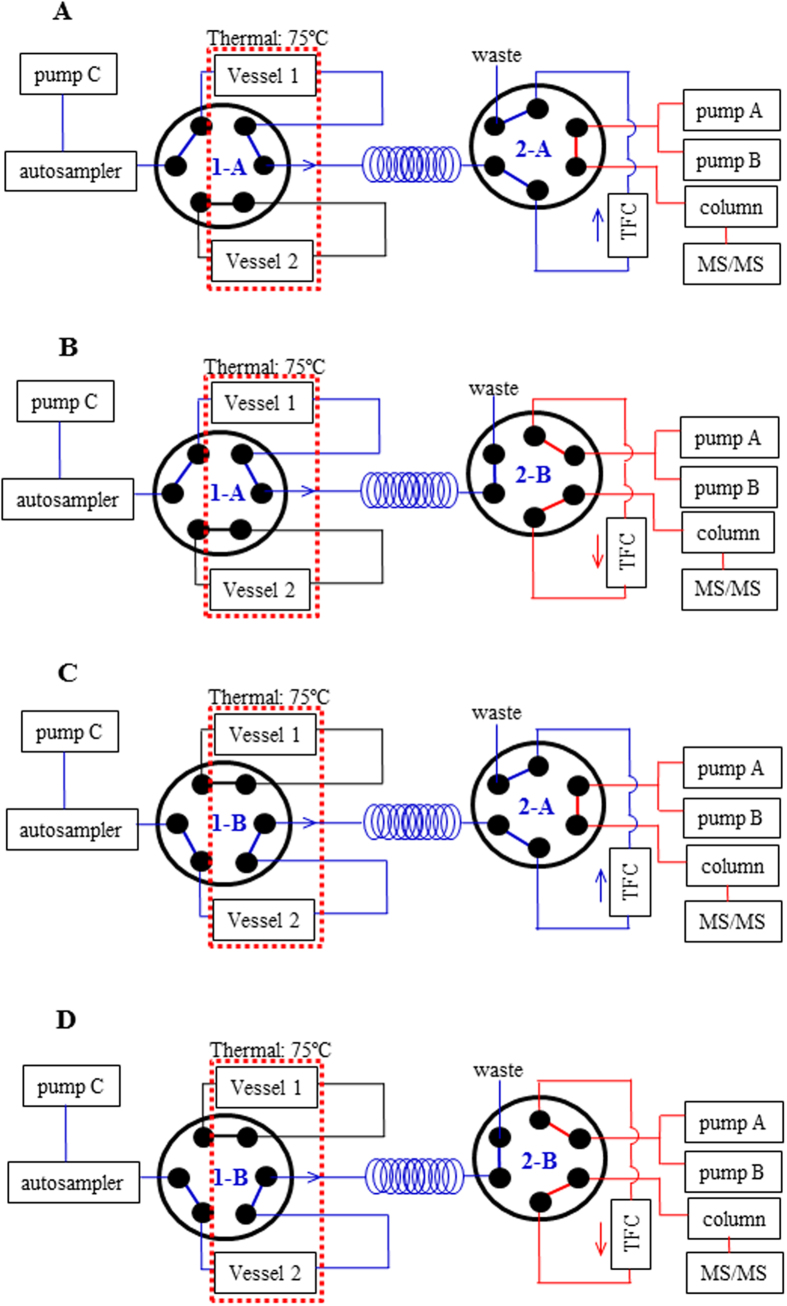
Schematic diagram of the online PWWE-TFC-LC-MS/MS platform. (**A**) extraction phase for Vessel **1,** and both valves were maintained at A-channel (1-A and 2-A); (**B**) elution phase for Vessel **1,** and Valves **1** and **2** were maintained at A-channel and B-channel (1-A and 2-B), respectively; (**C**) extraction phase for Vessel **2,** and Valves **1** and **2** were maintained at B-channel and A-channel (1-B and 2-A), respectively; (**D**) elution phase for Vessel **1,** and both valves were maintained at B-channel (1-B and 2-B). Detailed descriptions are included in the section “Configuration of the online PWWE-TFC-LC-MS/MS system”.

Because the PWWE module was composed of several LC units, it is easy to hyphenate it with LC-MS/MS by employing an online SPE technique. However, it remains a challenge to extract compounds from fluids at high flow rates. Fortunately, a TFC-based column could fulfill the requirement to retain small molecules regardless of their polarity. Theoretically, when large particles (approximately 50–100 μm in diameter) are used, the turbulence inside the TFC column (flow rate, >1.5 mL/min) allows the efficient removal of macromolecules because the larger molecules do not have time to diffuse into the pores of the particles and to interact with the stationary phase chemistry at a high flow rate, whereas small molecules (usually, <1500 Da), which do have time to diffuse into and out of the pores, are trapped by the column pores^20^. In addition, the lifespan of a TFC column is usually significantly longer than that of a conventional online SPE column. Typically, for the direct injection of biological samples, a TFC column can tolerate approximately 2000 injections of complex matrices without a significant decrease of column efficiency^21^. Therefore, TFC technology was introduced herein to extract analytes from the PWWE extract. Similarly to previous setups^22^,^23^,^24^,^25^, two electronic valves were employed to connect the different modules and to switch either vessel between the extraction and elution phases. Thus, an online PWWE-TFC-LC-MS/MS system was designed and configured as shown in Fig. 2.

As the key elements, the TFC and analytical columns were carefully assessed using 14 authentic compounds as the indicators. Firstly, satisfactory retention of some analytes, *e.g.*, sibiricose A_5_ and sibiricose A_6_, could not be achieved using conventional SPE columns with octadecyl silane particles (particle size, 5 μm); in contrast, the TFC column could retain all the 14 indicators. Then, a TurboFlow Cyclone column was selected because of its wider retention range compared with those of the other TFC candidates with identical size, such as Cyclone-P, Cyclone MCX, Cyclone MCX-2, and C_18_-P XL columns (all Thermo-Fisher products). Regarding the analytical column, a core-shell type column was superior to conventional columns packed with porous spherical octadecyl silane-modified particles, *e.g.*, Synergi Polar-RP 100A column (2.0 mm I.D. × 100 mm, 2.5 μm, Phenomenex) because of its greater separation efficiency, lower solvent consumption and faster separation time. Furthermore, an ACE UltraCore 2.5 SuperC_18_ column was selected from several core-shell type candidates because of its satisfactory characteristics in terms of back-pressure tolerance, peak capacity, and peak shape, in comparison with the Kinetex-C_18_ shell (2.1 mm I.D. × 100 mm, 2.6 μm, Phenomenex) and Capcell core ADME (2.1 mm I.D. × 150 mm, 2.7 μm, Shiseido, Tokyo, Japan) columns. In addition, mass spectrometric parameters were manually optimized for the quantitative analysis by introducing each pure compound into the Qtrap-MS individually. Optimum precursor-to-product ion transitions, DPs, and CEs for all analytes are shown in Table S1 (Supplemental information).

### Optimization of extraction conditions

The flow rate of the extraction solvent, the length of the PEEK tube, and the temperature played dominant roles for the performance of the online PWWE extraction. Increasing the temperature and the pressure, which is governed mainly by the flow rate and the tube dimensions, could definitely improve the extraction capacity; however, such increases are a challenge for the solvent delivery system and the connections between modules, as well as the ability of the column oven. Therefore, systematic evaluations were carried out by employing the 14 pure compounds as the indicators.

By running a series of assays in a temperature range of 50–85 °C with a step-size of 5 °C, the column oven temperature was ultimately set at 75 °C because higher temperatures could shorten the lifespans of both the column oven and TFC column, while lower temperatures could decrease extraction efficiency. For the compromise between flow rate and PEEK tube length, a flow rate of 2.5 mL/min was selected after a comparison among 1.0, 1.5, 2.0, 2.5, 3.0, and 3.5 mL/min, and the optimum length of PEEK tube was fixed at 1000 mm by careful comparisons among lengths of 800, 900, 1000, 1100 and 1200 mm. In addition, the extraction time was selected from a range of 1–15 min (step-size, 1 min) because the maximum value for the overall response was obtained after 3 min. These applied conditions could provide satisfactory extraction efficiency without sacrificing the lifespans of the apparatuses used.

Additionally, the optimal amount of crude materials was also evaluated. Given the outstanding extraction potential of PWWE and the high content of the analytes in Polygalae Radix, it was necessary to reduce the amount of sample used as much as possible. Although 0.5 mg of crude materials was utilized, some of the analytes still saturated the detector. Finally, the amount was fixed at 0.5 mg to ensure accurate weighing.

### Chemical profiling of Polygalae Radix using online PWWE-TFC-LC-MS/MS

The chemical profile of Polygalae Radix has been characterized in a previous study^26^, and the fragmentation patterns of the xanthones, sucrose esters, and triterpene saponins, which serve as the primary chemical categories of the compounds present, have been proposed^26^,^27^. Additionally, extensive phytochemical evaluations have also been reported^28^, and a number of compounds have been purified from this herbal medicine. Therefore, abundant information is available to assist in the structural assignment for the current study. The identification of the compounds was carried out mainly by applying the mass fragmentation rules and referring to the mass spectral information published in previous reports. A total of 62 components were detected in the extracts of Polygalae Radix using online PWWE-TFC-LC-IT-TOF-MS. Among these, the identities of 16 components were unambiguously determined by comparing their retention times and mass spectra with those of the pure compounds, while 41 components were tentatively identified and five could not be identified because of insufficient structural information. The retention times, mass spectral profiles, and assignments of all the compounds are presented in Table S2 (Supplemental information).

### Simultaneous determination of 14 analytes in Polygalae Radix using online PWWE-TFC-LC-MS/MS

**Figure 3:**
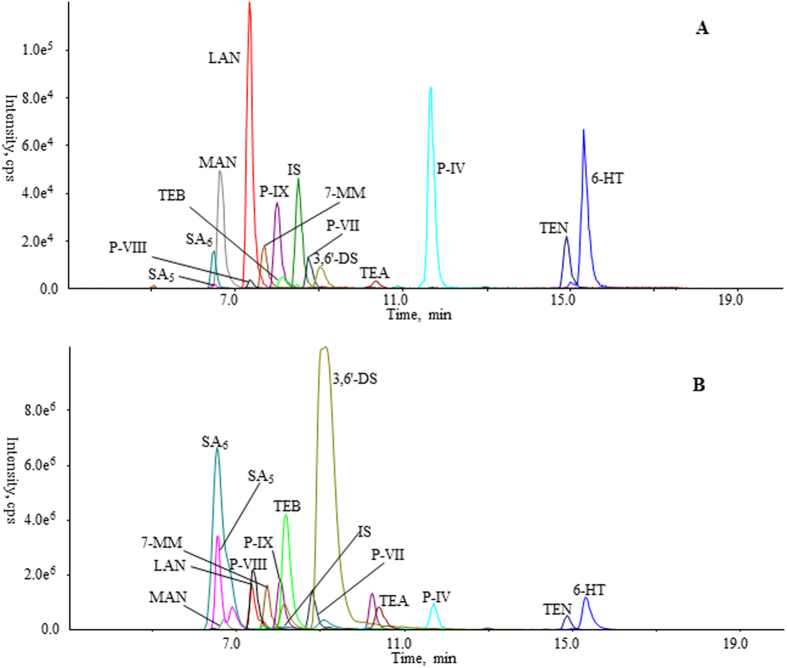
Representative overlaid extracted ion current (EIC) chromatograms of a mixed standard solution (**A**) and selected crude materials (PR4, B) by PWWE-TFC-LC-MS/MS. 6-HT, 6-hydroxy-1,2,3,7-tetramethoxyxanthone; LAN, lancerin; MAN, mangiferin; 7-MM, 7-*O*-methoxyl-mangiferin; SA_5_, sibiricose A_5_; SA_6_, sibiricose A_6_; P-IX, polygalaxanthone IX; P-IV, polygalaxanthone IV; P-VIII, polygalaxanthone VIII; P-VII, polygalaxanthone VII; TEB, tenuifoliside (**B**) TEN, tenuifolin; TEA, tenuifoliside A; 3,6′-DS, 3,6′-disinapoyl sucrose.

The developed online PWWE-TFC-LC-MS/MS system was validated using various assays. Representative chromatograms are shown in Fig. 3.

The coefficients of determination (R^2^) of the calibration curves in all the inter-run experiments were higher than 0.99 over the concentration ranges investigated (Table 1). The lower limits of quantitation (LLOQs) of all the analytes were lower than 32 ng/mL (0.64–32 ng/mL, Table 1), and their limits of detection (LODs) were less than 6.4 ng/mL (0.26–6.4 ng/mL, Table 1). It is noteworthy that the upper limits of quantification (ULOQs) of all the analytes were 4000 ng/mL except for sibiricose A_5_, polygalaxanthone VIII, tunuifoliside B, and 3,6′-disinapoyl sucrose. All these values suggested a satisfactory performance of the developed method regarding linearity and sensitivity.

The relative standard deviations (RSDs%) of the intra- and inter-day precisions were found to be lower than 8.36% and 8.32% (Table 2), respectively, for all the low, medium, and high concentration levels. The repeatability (RSDs% lower than 7.32% except those that exceeded the ULOQs) also meets the quantitative criteria for the simultaneous determination in complex matrices. Table 2 presents the results for the recovery, which is a key factor to assess the reliability of the developed method, and the values shown (recovery 85.82–114.6% with RSDs% in the range of 1.06–14.73%) indicate that the developed method is accurate.

Thus, the results demonstrate that the proposed method is sensitive, precise, accurate, and reproducible. Subsequently, the validated method was applied to the simultaneous quantitation of 14 analytes in PR1–10, and the results are summarized in Table 3. Among the 14 analytes, the content of sibiricose A_5_, sibiricose A_6_, polygalaxanthone VII, tenuifoliside A, and tenuifoliside B was beyond their respective linearity ranges.

To cross-validate the quantitative results obtained in the current study, two well-developed extraction protocols that are documented for Polygalae Radix in the Chinese Pharmacopeia^29^ were used with some minor modifications. Briefly, an aliquot (1.0 g) of each batch (PR1–10) was suspended in 50 volumes (g/v) of 70% aqueous methanol. Afterwards, each mixture was either ultrasonicated for 30 min or refluxed for 60 min to produce two types of extracts. All the extracts (20 samples in total) were then centrifuged at 10 000 × *g* for 10 min, and the obtained supernatants were individually subjected to conventional LC-Qtrap-MS analysis. Both the mass spectrometric parameters and gradient elution program were identical to those mentioned in the previous section “Simultaneous determination of 14 analytes in Polygalae Radix.” The validity of the conventional LC-Qtrap-MS assays was confirmed in terms of LOD, LLOQ, ULOQ, precision, repeatability, and recovery (data not shown) using the protocols published in the literature^22^. Representative chromatograms for mixed standard solutions, sonication extracts, and reflux extracts are shown in Fig. S2 (Supplemental information). Although only a limited volume of an environmentally sound solvent (warm water) was utilized, the quantitative results of the newly developed system were consistent with those of the traditional extraction methods, which indicates that the online PWWE-TFC-LC-MS/MS platform is reliable for the simultaneous determination of 14 analytes in Polygalae Radix.

## Discussion

Conventional analytical strategies usually involve labor intensive sample preparation procedures, such as sonication, centrifugation, concentration, reconstitution, filtration, and distribution into vials, which result in an additional sample preparation time of approximately two hours and the use of at least tens of milliliters of organic solvent. Moreover, it is challenging to stabilize oxygen- and light-sensitive compounds using the conventional approaches because of the long exposure time to air and/or light. However, these shortcomings of the routine techniques can be overcome by online PWWE-TFC-LC-MS/MS. The developed automated sample preparation process can be performed in three minutes and consumes only 0.5 mg of crude materials and 7.5 mL of water. The shorter extraction time and the use of a hermetically sealed vessel improve the stabilization of the analytes, and no specialized equipment is required. However, the effect of high temperature on thermo-sensitive components remains a challenge that requires attention. Thus, the newly configured system is advantageous in decreasing the analysis time, laboriousness, consumption of solvents and materials, and complexity of the instrumentation, all of which are consistent with “green analytical chemistry” theory.

Moreover, the newly constructed system is a versatile and flexible platform, which can be modified to match different objectives. If another pump is not available, modifications can be carried out as shown in the schematic in Fig. S3 (Supplemental information) so that only one extraction vessel is involved. Furthermore, two 6-channel/6-port valves can replace the two 2-channel/6-port valves to improve the throughput of online PWWE-TFC-LC-MS/MS (Fig. S4, Supplemental information), which shows that a single placement of the extraction vessels could result in parallel extraction for six different samples. Pressurized hot water extraction (PHWE), which uses a condensed phase of water at a temperature range from 100 °C (boiling point of water) to 374 °C (critical point of water), has been the most important of current sustainable extraction strategies^7^,^10^,^30^,^31^,^32^,^33^. Using the developed system, it is a simple task to achieve conditions for PHWE with only a couple of minor modifications, such as upgrading the column oven with more advanced equipment that is capable of reaching temperatures up to the critical point of water and replacing the membranes in the extraction vessels with specialized materials that permit the use of high temperatures. When targeted analysis is preferred, a selected online SPE column or other specialized column can be substituted for the TFC column to enhance the specificity of the system. In addition, the developed PWWE module can be employed alone as an efficient extraction tool, and this scalable module is also convenient for constructing a scale-up extraction system when large quantities of natural products are required to be efficiently extracted from plants.

Many types of crude materials are very precious, even more expensive than gold, *e.g.*, *Cordyceps sinesis*, *Stigma Croci*, *Ganoderma*, and wild *Panax ginseng*; hence, it is costly and difficult to collect enough material to meet the requirements of conventional extraction approaches. However, the newly configured system requires lower quantities of material to accomplish chemical fingerprinting because most of the analytes extracted by thermal water (75 °C) can be loaded onto an analytical column and subsequently transferred into a mass spectrometer. In addition, only a small amount of water, 7.5 mL in the current case, was consumed in a single extraction, which indicates substantial savings in solvent use. Recently, dried blood spots (DBSs) draw increasing attention as a convenient sampling approach in many areas, such as clinical diagnosis and drug monitoring. However, transferring DBS into biofluids prior to bioassays remains tedious. With the newly developed online PWWE-TFC-LC-MS/MS system, the DBSs can be easily analyzed by directly loading DBSs into the extraction vessels without any pretreatment. The developed analytical platform has the potential to be an ideal analytical tool for the chemical analysis of solid biological samples, *e.g.*, fecal samples, and the only pretreatment procedure required is pulverization.

In conclusion, a fully automated online PWWE-TFC-LC-MS/MS system was designed and configured for the first time. This system aims to fulfill the principles of “green analytical chemistry” by integrating online sample extraction, SPE, coupled with detection by mass spectrometry. Firstly, the PWWE module was constructed based on the phenomenon that a high backpressure is generated at high flow rates of water in a long narrow tube. A satisfactory extraction efficiency was demonstrated for the PWWE module in comparison with validated protocols. Then, a TFC column was used as the interface between the PWWE module and the LC-MS/MS, and two electronic 2-channel/6-port valves were used to alternate the two parallel extraction vessels between the extraction and elution phases. The newly developed platform was applied to the qualitative and quantitative analyses of the components of Polygalae Radix. The findings demonstrate that the online PWWE-TFC-LC-MS/MS analytical platform decreases analysis time, assay laboriousness, and solvent and material consumption and is straightforward to construct. Therefore, the versatile and automated system offers a promising choice for the direct chemical analysis of plants and other solid samples by following the theory of “green analytical chemistry.”

## Methods

### Chemicals and materials

Fourteen pure compounds, including 3,6′-disinapoyl sucrose, 6-hydroxy-1,2,3,7-tetramethoxyxanthone, lancerin, mangiferin, 7-*O*-methoxylmangiferin, polygalaxanthone IV, polygalaxanthone VII, polygalaxanthone VIII, polygalaxanthone IX, sibiricose A_5_, sibiricose A_6_, tenuifolin, tenuifoliside A, and tenuifoliside B, as well as liquiritin, that served as an internal standard (IS) were obtained from the chemical library of State Key Laboratory of Natural and Biomimetic Drugs, Peking University (Beijing, China). Their chemical structures were further confirmed by ^1^H- and ^13^C-NMR spectra, and the purity of each reference compound was determined to be greater than 98% using LC-IT-TOF-MS (Shimadzu, Tokyo, Japan).

Formic acid, methanol, ACN, and dimethylsulfoxide (DMSO) were of LC-MS grade and purchased from Thermo-Fisher (Pittsburgh, PA, USA). Deionized water was prepared in-house using a Milli-Q Integral water purification system (Millipore, Bedford, MA, USA). The other chemicals were of analytical grade and commercially supplied by Beijing Chemical Works (Beijing, China).

Ten batches of Polygalae Radix (PR1–10, Table 3) were collected from different habitats in China, and their botanical origins were authenticated as the dried roots of *Polygala tenuifolia* Willd. All the voucher specimens are deposited in the herbarium of the Modern Research Center for Traditional Chinese Medicine, Beijing University of Chinese Medicine (Beijing, China).

### Apparatus

Several Shimadzu LC units (Tokyo, Japan), including an online vacuum degasser (DGU-20A3R), three pumps (LC-20AD_XR_), an auto-sampler (SIL-20AC_XR_), a column oven (0–85 °C, CTO-20 A), two electronic 2-channel/6-port valves (FCV-12AH), and a controller (CBM-20 A), were used. An ABSciex 5500 Qtrap mass spectrometer (Foster City, CA, USA) equipped with a Turbo V^TM^ electronic spray ionization (ESI) interface enabled quantitative measurements, while a Shimadzu IT-TOF-MS mounted ESI source was used for high-resolution mass spectrometry.

### Sample preparation

The pure compounds were dissolved in DMSO to prepare stock solutions at a concentration of 4 mg/mL. Then, stock solutions of all the compounds were pooled and diluted to the desired concentration levels with 50% aqueous methanol containing an IS (final concentration, 45 ng/mL) to obtain a series of mixed standard solutions.

The crude materials (PR1– 10) were dried using a universal oven with forced convection (FD115, Tuttlingen, Germany) at 40 °C for 4 days. Each dried sample was pulverized using a sample mill (model YF102, RuianYongli Pharmacy Machinery Company, Jiangsu, China), and the resulting powders were then sieved through a 24 mesh sieve (0.85 mm, I.D.).

### Construction of the PWWE module and assessment of the extraction efficiency

As the core unit of the new hybrid platform, the PWWE module was composed of a solvent delivery unit, a column oven, and an extraction vessel (a hollow guard column, 3.0 mm I.D. ×4.0 mm, Phenomenex, Torrance, CA, USA), as well as steel and PEEK tubes (Fig. 1). An accurately weighed (approximately 0.5 mg) amount of a selected sample powder (PR4) was thoroughly mixed with clean diatomaceous earth, and the hollow guard column was filled with the mixture. After being sealed with two filter membranes (0.22 μm) and two caps, the vessel was then placed into a matching cartridge holder (Phenomenex) (Fig. 1). The pump enabled the delivery of water at a flow rate of 2.5 mL/min for 3 minutes. The extraction vessel was maintained at 75 °C in the column oven. A steel tube (0.30 mm I.D. ×200 mm) connected the vessel to the pump and was stored in the column oven to efficiently warm the solvent, whereas a long PEEK tube (0.13 mm I.D. ×1000 mm) was connected to the end of the vessel to generate the desired back pressure (approximately 13 MPa) and placed out of the oven to cool down the thermal effluent from the vessel. The fluid from the PWWE module was collected at the outlet of the long PEEK tube, concentrated, and reconstituted with 70% aqueous methanol to yield the PWWE extract. Because ultrasound-assisted extraction with 70% aqueous methanol has been confirmed to extract the active components of Polygalae Radix by the Chinese Pharmacopoeia^29^, this routine technique was used to assess the extraction efficiency of PWWE. Because water served as the extraction solvent for PWWE, water sonication was also used for the evaluation. Additionally, a reflux protocol documented in the Chinese Pharmacopoeia^29^ was also evaluated in the assessment. A portion of the extract produced by sonication, reflux, or PWWE, which corresponded to an equivalent amount of the crude material, was injected into an LC-IT-TOF-MS (Shimadzu) equipped with an ACE Ultra-Core 2.5 SuperC18 column (3.0 mm I.D. × 150 mm, 2.5 μm, Advance Chromatography Technologies Ltd., Aberdeen, Scotland). A gradient elution with 0.1% aqueous formic acid (A) and ACN (B) was programmed as follows: 0–3 min, 10% B; 3–10 min, 10–20% B; 10–25 min, 20–30% B; 25–35 min, 30–45% B; 35–40 min, 45–60% B; 40–45 min, 60–90% B; 45–48 min, 90% B; 48–48.1 min, 90–10% B; 48.1–60 min, 10% B; flow rate, 0.3 mL/min. Default parameters were applied for the IT-TOF-MS domain, and the data acquisition and analysis were performed using the Shimadzu LCMS Solution Version 3 software.

### Configuration of the online PWWE-TFC-LC-MS/MS system

To online hyphenate PWWE, TFC, and LC-MS/MS, two electronic 2-channel/6-port valves (Valves **1** and **2**) were employed to connect these three parts. A brief schematic of the entire system is illustrated in Fig. 2. Two parallel extraction vessels were used to guarantee the throughput, and an auto-sampler was employed to initiate a single extraction-elution cycle and synchronize the entire system *via* a starting signal. Pumps A and B delivered the mobile phase to the analytical column, whereas pump C supplied the solvent for the PWWE module. The two electronic valves were responsible for alternating either vessel between the extraction and elution phases. The TFC column (TurboFlow Cyclone column, 1.0 mm I.D. × 50 mm, Thermo Fisher Scientific Inc., Rockford, IL, USA) was used to universally trap components with relatively low molecular weight (usually < 1500 Da) while expelling macromolecules, *e.g.*, proteins and polysaccharides, to avoid contamination at the ESI interface. To prevent peak broadening, the TFC column alternated between forward and reverse elution by switching Valve **2** between channels A and B. The chromatographic separation was conducted on an ACE Ultra-Core 2.5 SuperC18 column. The IT-TOF-MS or Qtrap-MS instruments detected the analytes that exited from the analytical column. The ABSciex Analyst Software package Version 1.6.2 was used for quantitative data acquisition and processing, whereas the Shimadzu LCMS Solution software was utilized to process the qualitative data.

### Qualitative characterization

After loading a selected sample (PR4, approximately 0.5 mg) that was completely dispersed with diatomaceous earth into Vessel **1**, the auto-sampler was used to trigger the extraction and subsequent measurement by the injection of 2 μL of 50% aqueous methanol. Each measurement was divided into two phases (extraction phase, 0–3 min; elution phase, 3–63 min) by switching Valve **2** at 3 min (channel A → channel B), while Valve **1** was always maintained at channel A to preserve the connection between the auto-sampler and Vessel **1** (Fig. 2A,B). Pure water was supplied by pump C at a flow rate of 2.5 mL/min during the extraction phase (0–3 min), whereas pumps A and B delivered 0.1% aqueous formic acid (A) and ACN (B), respectively, with a gradient program as follows: 0–3 min, 0% B; 3–6 min, 10% B; 6–13 min, 10–20% B; 13–28 min, 20–30% B; 28–38 min, 30–45% B; 38–43 min, 45–60% B; 43–48 min, 60–90% B; 48–51 min, 90% B; 51.1–63 min, 0% B; flow rate, 0.3 mL/min. An IT-TOF-MS instrument was used for high-resolution mass spectrometry, and parameters following the description in the literature were applied for the MS domain^34^.

### Simultaneous determination of 14 analytes in Polygalae Radix

Following the introduction of the accurately weighed crude materials (PR1–10, approximately 0.5 mg for each) and diatomaceous earth into the extraction vessels (two batches at a time), extraction was initiated by the auto-sampler. Valve **1** was switched at 0 min to choose vessel, whereas Valve **2** (channel A → channel B) was used at 3 min to split each run into extraction (Fig. 2A,C) and elution phases (Fig. 2B,D) for either vessel. The injection volume of 50% aqueous methanol with the IS (final concentration, 45 ng/mL) was set at 2.0 μL. Pure water was supplied by pump C at a flow rate of 2.5 mL/min during extraction phase, whereas the mobile phase consisting of 0.1% aqueous formic acid (A) and ACN (B) was delivered following a gradient program as follows: 0–3 min, 0% B; 3–3.01 min, 0–20% B; 3.01–15 min, 20–45% B; 15–15.01 min, 45–0% B; 15.01–27 min, 0% B; flow rate, 0.3 mL/min.

The curtain gas (CUR) and two source gases (GS1 and GS2) were fixed at pressures of 20, 35, and 35 psi, respectively. The source temperature was set at 500 °C. The negative ionization polarity was used, and the sprayer voltages were fixed at −4500 V. Two precursor-to-product ion transitions were recorded for each analyte or IS. The information concerning precursor-to-product ion transitions, optimized declustering potential (DP), and collision energy (CE) is shown in Table S1 (Supplemental information), while the dwell time, entrance potential (EP), and collision cell exit potential (CXP) levels of each ion transition were fixed at 50 ms, 10 V, and 16 V, respectively.

In comparison with the conventional protocols described in the literature^22^,^35^, some modifications were carried out for the method validation assays. Briefly, regarding linearity, LLOQ, and LOD, as well as intra- and inter-day assays, the crude materials in the extraction vessel were replaced with equivalent amounts of clean diatomaceous earth, and aliquots (2.0 μL) of the mixed standard solutions described above (serial concentration levels for linearity, LOD, LLOQ, and ULOQ assays, and low, medium, and high concentration levels for intra- and inter-day assays) were individually injected by the auto-sampler. For the repeatability assay, accurately weighed (approximately 0.5 mg) crude materials were randomly sampled from a selected batch (PR-4) in six replicates, and individual measurements were then performed using PWWE-TFC-LS-MS/MS. For each measurement the extraction was initiated by injecting 2.0 μL of 50% aqueous methanol with the IS. In contrast to conventional extraction methods, a modified approach was performed to assess the recovery. Internal standards were added to solutions with low (160 ng/mL), medium (800 ng/mL), and high (3000 ng/mL) concentration levels of the mixed standards, and the resulting mixtures were spiked into extraction vessels that were filled with crude materials (PR-4) and clean diatomaceous earth using auto-sampler (2.0 μL). The recovery was calculated according to the following formula: recovery (%) =(measured-background)/added ×100. Because each sample was extracted and measured online, the stability assay was therefore inapplicable to the current case. The LOD was determined with a signal to noise (S/N) ratio >3, LLOQ was determined with S/N >10 and <20% of the coefficient of variation (CV, standard deviation divided by the mean), and ULOQ was determined with the intensity level below the detector saturation that corresponds to the highest concentration of each calibration curve. All the reproducibilities were expressed as RSDs%.

## Additional Information

**How to cite this article**: Song, Y. *et al.* Chromatographic analysis of Polygalae Radix by online hyphenating pressurized liquid extraction. *Sci. Rep.* **6**, 27303; doi: 10.1038/srep27303 (2016).

## Supplementary Material

Supplementary Information

## Figures and Tables

**Table 1:** Linear regression data, lower limits of quantification (LLOQs), and limits of detection (LODs) for all targeted analytes.

Compound	Calibration curves	*R*^*2*^	Range (ng/mL)	LOD (ng/mL)	LOQ (ng/mL)
6-Hydroxy-1,2,3,7-tetramethoxyxanthone	y = 0.816x − 0.0197	0.9986	16–4000	1.28	3.2
Lancerin	y = 0.831x + 0.222	0.9986	80–4000	0.96	2.24
Mangiferin	y = 0.596x + 0.301	0.9988	80–4000	1.28	3.2
7-*O*-Methoxyl-mangiferin	y = 0.213x + 0.0631	0.9978	80–4000	3.2	6.4
Sibiricose A_5_	y = 0.018x − 0.00874	0.9988	160–20000	6.4	16
Sibiricose A_6_	y = 0.153x − 0.00507	0.9976	32–4000	0.64	1.28
Polygalaxanthone IX	y = 0.42x + 0.206	0.9982	80–4000	0.64	1.28
Polygalaxanthone IV	y = 0.902x + 0.511	0.9980	80–4000	0.26	0.64
Polygalaxanthone VIII	y = 0.0331 − 0.00696	0.9994	160–100000	4.8	6.4
Polygalaxanthone VII	y = 0.138x + 0.0226	0.9998	80–4000	1.28	2.24
Tenuifoliside B	y = 0.0994x + 0.0101	0.9968	80–10000	1.28	3.2
Tenuifolin	y = 0.294 − 0.000497	0.9980	6.4–4000	0.96	3.2
Tenuifoliside A	y = 0.048x + 0.0222	0.9984	80–4000	6.4	32
3,6′-Disinapoyl sucrose	y = 0.166x − 0.135	0.9998	160–100000	1.28	3.2

**Table 2:** Intra- and inter-day assay results (RSD%) of quality control samples (high, medium, and low concentration levels), and recovery assay (high, medium, and low concentration levels) results for all targeted analytes.

Analyte	Intra-day RSD (%,*n* = 6)			Inter-day RSD (%, *n* = 6)			Recovery (%, *n* = 3)					
	Low (160 ng/mL)	Medium (800 ng/mL)	High (3000 ng/mL)	Low (160 ng/mL)	Medium (800 ng/mL)	High (3000 ng/mL)	Low		Medium		High	
							Mean	RSD	Mean	RSD	Mean	RSD
6-Hydroxy-1,2,3,7-tetramethoxyxanthone	4.12	4.50	2.36	5.01	6.46	1.89	85.82	5.67	97.83	10.39	114.6	11.45
Lancerin	4.00	2.96	4.10	5.12	1.64	2.65	86.85	11.43	89.92	9.95	95.05	12.93
Mangiferin	3.12	3.54	4.51	2.59	1.83	3.86	91.61	3.52	90.20	12.37	95.46	13.30
7-*O*-Methoxyl-mangiferin	4.90	3.55	2..86	4.02	3.11	3.60	106.1	30.77	109.5	13.46	88.60	9.38
Sibiricose A_5_	7.88	4.41	2.98	4.49	4.10	3.12	N.Q.^a^	N.A.^b^	N.Q.	N.A.	N.Q.	N.A.
Sibiricose A_6_	3.58	3.40	3.88	3.80	4.37	5.27	N.Q.	N.A.	N.Q.	N.A.	N.Q.	N.A.
Polygalaxanthone IX	3.03	1.98	4.67	4.61	3.17	4.56	91.14	7.10	95.20	14.73	92.91	11.16
Polygalaxanthone IV	3.84	2.57	2.71	3.59	3.20	5.56	95.17	11.63	98.08	10.09	101.3	5.55
Polygalaxanthone VIII	4.88	2.83	4.96	4.59	3.57	4.68	89.30	14.12	107.1	1.60	103.51	5.36
Polygalaxanthone VII	3.16	2.25	1.68	3.72	1.30	5.70	N.Q.	N.A.	N.Q.	N.A.	N.Q.	N.A.
Tenuifoliside B	3.34	2.13	5.49	4.93	4.14	8.32	N.Q.	N.A.	N.Q.	N.A.	N.Q.	N.A.
Tenuifolin	8.36	4.80	4.53	5.21	3.89	5.32	99.77	9.43	108.57	11.62	106.0	1.06
Tenuifoliside A	7.05	4.96	3.54	5.29	7.10	5.36	N.Q.	N.A.	N.Q.	N.A.	N.Q.	N.A.
3,6′-Disinapoyl sucrose	3.90	5.00	3.48	3.47	5.68	5.35	N.Q.	N.A.	N.Q.	N.A.	N.Q.	N.A.

^a^N.Q., not quantifiable due to beyond upper limit of quantitation.

^b^N.A., not applicable.

**Table 3:** The contents of fourteen investigated compounds in ten batches of Polygalae Radix (PR1–PR10).

No.	Habitat	Content (μg/g)^a^													
		6-HT	LAN	MAN	7-MM	SA_5_	SA_6_	P-IX	P-IV	P-VIII	P-VII	TEB	TEN	TEA	3,6′-DS
PR-1	Shanxi	4.15	11.73	1.49	39.91	N.Q.^b^	N.Q.	22.00	5.69	406.22	N.Q.	N.Q.	11.82	N.Q.	866.67
PR-2	Shaanxi	12.19	13.00	3.87	1.04	N.Q.	N.Q.	31.76	7.67	474.29	N.Q.	N.Q.	11.62	N.Q.	961.90
PR-3	Hebei	14.49	14.98	1.36	51.43	N.Q.	N.Q.	25.47	7.31	551.02	N.Q.	N.Q.	9.67	N.Q.	885.71
PR-4	He’nan	13.71	16.25	2.82	61.25	N.Q.	N.Q.	30.38	8.08	545.83	N.Q.	N.Q.	9.96	N.Q.	833.33
PR-5	Sichuan	2.80	13.90	2.51	52.20	N.Q.	N.Q.	25.37	8.73	595.12	N.Q.	N.Q.	13.12	N.Q.	1112.20
PR-6	Anhui	12.51	23.79	4.89	101.70	N.Q.	N.Q.	68.09	13.15	948.94	N.Q.	N.Q.	27.83	N.Q.	855.32
PR-7	Inner Mongolia	10.87	14.87	2.83	58.70	N.Q.	N.Q.	39.48	9.70	513.04	N.Q.	N.Q.	10.61	N.Q.	726.09
PR-8	Liaoning	20.18	25.33	5.87	118.67	N.Q.	N.Q.	64.44	11.33	991.11	N.Q.	N.Q.	23.42	N.Q.	893.33
PR-9	Heibei	5.40	18.65	5.67	93.95	N.Q.	N.Q.	58.14	15.95	888.37	N.Q.	N.Q.	25.95	N.Q.	352.09
PR-10	Shanxi	3.84	18.31	3.02	94.22	N.Q.	N.Q.	31.78	5.91	755.56	N.Q.	N.Q.	13.87	N.Q.	853.33

^a^6-HT, 6-hydroxy-1,2,3,7-tetramethoxyxanthone; LAN, lancerin; MAN, mangiferin; 7-MM, 7-*O*-methoxy-mangiferin; SA_5_, sibiricose A_5_; SA_6_, sibiricose A_6_; P-IX, polygalaxanthone IX; P-IV, polygalaxanthone IV; P-VIII, polygalaxanthone VIII; P-VII, polygalaxanthone VII; TEB, tenuifoliside B; TEN, tenuifolin; TEA, tenuifoliside A; 3,6′-DS, 3,6′-disinapoyl sucrose.

^b^N.Q., not quantifiable due to greater than the upper limit of quantitation.
