# Supplementary material for: Chromatographic analysis of Polygalae Radix by online hyphenating pressurized liquid extraction
Source: Sci Rep. 2016 Jun 7;6:27303. doi: 10.1038/srep27303 (PMC4895155; doi:10.1038/srep27303)
Supplement: Supplementary Information [file srep27303-s1.doc]

**Supplemental information**

**Chromatographic analysis of Polygalae Radix by online hyphenating pressurized liquid extraction**

Yuelin Song ^1,#^, Qingqing Song ^1,2,#^, Jun Li ^1^, Shepo Shi ^1^, Liping Guo ^3^, Yunfang Zhao ^1^, Yong Jiang ^4^* & Pengfei Tu ^1^*

^1^ Modern Research Center for Traditional Chinese Medicine, Beijing University of Chinese Medicine, Beijing 100029, China, ^2^ School of Chinese Materia Medica, Beijing University of Chinese Medicine, Beijing 100102, China, ^3^ Thermo-Fisher Scientific Corporation, Shanghai 201205, China, ^4^ State Key Laboratory of Natural and Biomimetic Drugs, School of Pharmaceutical Sciences, Peking University, Beijing 100191, China.

^#^ These two authors contributed equally to this article.

Correspondence and requests for materials should be addressed to Y. J. (yongjiang@bjmu.edu.cn) or P.-F. T. (pengfeitu@163.com)

**Table S1** Retention times (*t*_R_), MS^1^ and MS^2^ spectral information, and compound-dependent mass parameters for fourteen analytes and internal standard (IS)

| Analyte | *t*_R_ (min) | MRM* | DP (V) | CE (V) |
| --- | --- | --- | --- | --- |
| 6-Hydroxy-1,2,3,7-tetramethoxyxanthone | 15.33 | **331> 301;** 331> 316 | -180 | **-28;** -24 |
| Lancerin | 7.36 | **405****> 257;** 405> 285 | -160 | **-44;** -33 |
| IS | 8.56 | **417> 255;** 417> 135 | -130 | **-26;** -40 |
| Mangiferin | 6.67 | **421> 331;** 421> 301 | -140 | **-31;** -31 |
| 7-*O*-Methoxyl-mangiferin | 7.73 | **435> 345;** 435> 315 | -140 | **-30;** -30 |
| Sibiricose A_5_ | 6.51 | **517> 193;** 517> 175 | -190 | **-31;** -32 |
| Sibiricose A_6_ | 6.50 | **547> 205;** 583> 547 | -200 | **-31;** -23 |
| Polygalaxanthone IX | 8.03 | **551> 243;** 587> 551 | -130 | **-36;** -24 |
| Polygalaxanthone IV | 11.69 | **565> 257;** 565> 242 | -160 | **-34;** -70 |
| Polygalaxanthone VIII | 7.39 | **567> 447;** 567> 345 | -130 | **-38;** -42 |
| Polygalaxanthone VII | 8.80 | **611> 303;** 647> 611 | -130 | **-42;** -25 |
| Tenuifoliside B | 8.18 | **667> 461;** 667> 205 | -200 | **-34;** -37 |
| Tenuifolin | 14.87 | **679> 455;** 679> 425 | -70 | **-38;** -48 |
| Tenuifoliside A | 10.38 | **717> 681;** 681> 443 | -100 | **-25;** -34 |
| 3,6'-Disinapoyl sucrose | 9.06 | **753> 205;** 789> 753 | -200 | **-39;** -26 |

*: Precursor-to-product ion transitions in bold and their corresponding collision energy (CE) in bold are selected for quantitative analysis.

**Table S2** The retention times, mass spectrometric information and plausible identities of the components detected from Polygalae Radix

| No. | *t*_R_ (min) | MS^1^ | Molecular formula | MS^2^ | Identity | Ref. |
| --- | --- | --- | --- | --- | --- | --- |
| 1 | 2.893 | 537.1189 | C_24_H_26_O_14_ | 387.07; 315.05; 267.03; 107.43 | sibiricaxanthone A or sibiricaxanthone B | [1] |
| 2 | 2.893 | 517.1519 | C_22_H_30_O_14_ | 337.08; 193.05; 175.04 | sibiricose A_5_ ^#^ | [1] |
| 3 | 3.285 | 547.1634 | C_23_H_32_O_15_ | 367.09; 223.06; 205.05 | sibiricose A_6_ ^#^ | [1] |
| 4 | 4.423 | 405.0793 | C_19_H_18_O_10_ | 285.04; 257.04 | neolancerin | [2] |
| 5 | 4.492 | 537.1348 | C_24_H_26_O_14_ | 453.14; 315.05; 267.03; 206.39 | sibiricaxanthone A or sibiricaxanthone B | [1] |
| 6 | 8.813 | 511.2880 | - | 496.26; 481.24 | unknown | - |
| 7 | 8.510 | 405.0750 | C_19_H_18_O_10_ | 315.05; 285.04; 257.04 | lancerin ^#^ | [3] |
| 8 | 8.510 | 421.0642 | C_19_H_18_O_11_ | 403.06; 331.04; 301.03 | mangiferin ^#^ | [4] |
| 9 | 10.043 | 435.0885 | C_20_H_20_O_11_ | 345.06; 315.05; 272.03 | 7-*O*-methylmangiferin ^#^ | [3] |
| 10 | 10.200 | 561.1761 | C_24_H_34_O_15_ | 323.09; 237.08 | sibiricose A_2_ or glomeratose A | [1] |
| 11 | 10.670 | 567.1227 | C_25_H_28_O_15_ | 447.09; 345.05; 315.04; 272.03 | polygalaxanthone III or polygalaxanthone XI | [1,3] |
| 12 | 11.737 | 567.1366 | C_25_H_28_O_15_ | 447.08; 315.05; 297.0309;272.03 | polygalaxanthone VIII ^#^ | [3] |
| 13 | 11.972 | 667.1809 | C_30_H_36_O_17_ | 529.15; 461.12; 367.10; 299.07 | tenuifoliside B ^#^ | [5] |
| 14 | 12.578 | 753.2189 | C_34_H_42_O_19_ | 547.16; 367.10; 277.08 | sibiricose A_4_ | [1] |
| 15 | 12.875 | 637.1689 | C_29_H_34_O_16_ | 461.12; 443.11; 323.08 | sibiricaxanthone F | [6] |
| 16 | 13.072 | 667.1793 | C_30_H_36_O_17_ | 529.15; 461.12; 353.08; 299.07; 239.06 | tenuifoliside B ^#^ | [5] |
| 17 | 13.710 | 799.2309 | C_35_H_44_O_21_ | 753.22; 277.82 | sibiricaxanthone G | [6] |
| 18 | 14.073 | 561.1818 | C_24_H_34_O_15_ | 323.10; 237.07 | sibiricose A_2_ or glomeratose A | [1] |
| 19 | 14.425 | 611.1646 | C_27_H_32_O_16_ | 303.05; 273.00; 245.01 | polygalaxanthone VII ^#^ | [7] |
| 20 | 14.572 | 581.1496 | C_26_H_30_O_15_ | 375.06; 273.04; 258.01 | polygalaxanthone V | [7] |
| 21 | 15.748 | 753.2167 | C_34_H_42_O_19_ | 573.14; 547.16; 367.10; 325.09 | 3,6'-disinapoyl sucrose ^#^ | [5] |
| 22 | 16.178 | 723.2051 | C_33_H_40_O_18_ | 547.16; 529.15; 517.15; 499.14; 337.09; 265.07 | (3-*O*-feruloyl)-*β*-D-fructofuranosyl-(6-*O*-sinapoyl)-*α*-D-glucopyranoside | [8] |
| 23 | 16.833 | 651.1859 | C_30_H_36_O_16_ | 499.13; 443.11; 281.06; 239.05 | [3-*O*-(3,4-dimethoxycinnamoyl)]-*β*-D-fructofuranosyl-(6-*O*-*p*-hydroxybenzoyl)-*α*-D-glucopyranoside | [9] |
| 24 | 17.578 | 551.1674 | C_25_H_28_O_14_ | 431.12; 285.07; 243.06 | polygalaxanthone IX ^#^ | [3] |
| 25 | 17.998 | 651.1865 | C_30_H_36_O_16_ | 529.15; 445.13; 367.10; 323.08; 223.06 | (3-*O*-sinapoyl)-*β*-D-fructofuranosyl-(6-*O*-benzoyl)-*α*-D-glucopyranoside | [9] |
| 26 | 18.155 | 621.1804 | C_29_H_34_O_15_ | 499.14; 337.09; 193.05 | reiniose C or reiniose B | [8] |
| 27 | 18.370 | 681.1971 | C_31_H_38_O_17_ | 529.15; 443.11; 367.10; 281.06 | tenuifoliside A ^#^ | [5] |
| 28 | 21.387 | 621.1661 | C_29_H_34_O_15_ | 499.14; 441.12; 337.07 | reiniose C or reiniose B | [8] |
| 29 | 21.457 | 765.2115 | C_35_H_42_O_19_ | 723.20; 705.20; 589.16; 529.13; 296.99 | (3-*O*-feruloyl-6-*O*-sinapoyl)-*β*-D-fructofuranosyl-(6-*O*-acetyl)-*α*-D-glucopyranoside | [9] |
| 30 | 22.198 | 767.2299 | C_35_H_44_O_19_ | 605.16; 529.15; 367.10 | tenuifoliside C | [5] |
| 31 | 22.610 | 1295.3997 | C_58_H_72_O_33_ | 1173.35; 1119.34; 997.30; 793.22; 689.20; 379.91 | tenuifoliose C | [10] |
| 32 | 22.372 | 565.1309 | C_27_H_32_O_15_ | 257.04; 242.02 | polygalaxanthone IV | [7] |
| 33 | 22.680 | 737.2279 | C_34_H_42_O_18_ | 615.18; 499.14; 467.13 | reiniose A | [8] |
| 34 | 23.300 | 711.2197 | C_32_H_40_O_18_ | 665.20; 279.99; 122.26 | telephiose C | [11] |
| 35 | 23.567 | 1307.4065 | C_59_H_72_O_33_ | 1265.38; 1161.35; 1119.35; 1039.32; 471.41; 293.75 | tenuifoliose I or tenuifoliose J | [12] |
| 36 | 23.717 | 1265.4132 | C_57_H_70_O_32_ | 1119.34; 1145.35; 999.30; 997.29; 876.26 | tenuifoliose K | [12] |
| 37 | 24.687 | 1337.4232 | C_60_H_74_O_34_ | 1277.39; 1161.35; 1119.34; 1039.32; 763.62; 584.94 | tenuifoliose B or tenuifoliose D | [10] |
| 38 | 25.220 | 611.1630 | C_27_H_32_O_16_ | 317.06; 302.04; 216.61 | polygalaxanthone VII | [7] |
| 39 | 25.900 | 1307.4084 | C_59_H_72_O_33_ | 1265.39; 1161.35; 1119.35; 1039.31 | tenuifoliose J or tenuifoliose I | [12] |
| 40 | 26.187 | 1103.5223 | C_53_H_84_O_24_ | 1073.52; 745.39; 539.34; 455.31; 425.30; 337.11 | polygalasaponin XXVIII ^#^ | [13] |
| 41 | 26.187 | 695.1930 | C_32_H_40_O_17_ | 543.16; 237. 07 | [3-*O*-(3,4,5-trimethoxycinnamoyl)]-*β*-D-fructofuranosyl-(6-*O*-*p*-anisoyl)-*α*-D-glucopyranoside | [5] |
| 42 | 26.977 | 1337.4231 | C_60_H_74_O_34_ | 1277.39; 1161.35; 1039.31 | tenuifoliose B or tenuifoliose D | [10] |
| 43 | 27.128 | 762.3196 (2) | C_70_H_110_O_36_ | 1158.57; 690.30 (2) | onjisaponin Te | [14] |
| 44 | 27.315 | 1349.4258 | C_61_H_74_O_34_ | 1227.37; 1203.37; 1143.34 | tenuifoliose H | [12] |
| 45 | 27.458 | 807.2302 | C_37_H_44_O_20_ | 765.21; 705.19; 631.18; 543.14; 349.08 | (3-*O*-feruloyl-6-*O*-sinapoyl)-*β*-D-fructofuranosyl-(4,6-di-*O*-acetyl)-*α*-D-glucopyranoside | [9] |
| 46 | 28.078 | 777.2122 | C_36_H_42_O_19_ | 735.20; 717.19; 601.17; 559.16 | (3,6-di-*O*-feruloyl)-*β*-D-fructofuranosyl-(4,6-di-*O*-acetyl)-*α*-D-glucopyranoside | [9] |
| 47 | 28.308 | 1379.464 | C_62_H_76_O_35_ | 1337.42; 1203.37; 1143.34; 1039.32 | tenuifoliose A | [10] |
| 48 | 29.435 | 331.0757 | C_17_H_16_O_7_ | 316.056; 301.03 | 6-hydroxy-1,2,3,7-tetramethoxyxanthone | [15] |
| 49 | 30.857 | 849.2319 | - | 807.22; 789.21; 673.20; 631.17 | unknown | - |
| 50 | 30.857 | 819.1965 | - | 777.1968; 643.1753; 601.1708 | unknown | - |
| 51 | 31.788 | 679.3612 | C_36_H_56_O_12_ | 455.31; 425.30 | tenuifolin ^#^ | [16] |
| 52 | 32.210 | 849.2355 | - | 807.22; 789.2127; 765.21; 673.18; 631.17; 613.16; 543.14; 349.08 | unknown | - |
| 53 | 34.453 | 287.0571 | C_15_H_12_O_6_ | 272.03; 257.01 | dihydroxy-dimethoxyxanthone | [17] |
| 54 | 34.643 | 938.3852 (2) | C_87_H_130_O_44_ | 866.85 (2); 857.35 (2); 762.82; 391.92 | onjisaponin Sg | [18] |
| 55 | 34.762 | 961.3891 (2) | - | 889.85 (2); 770.81 (2); 699.21; 679.36 | unknown | - |
| 56 | 35.640 | 891.2535 | C_41_H_48_O_22_ | 849.23; 807.23; 715.19; 673.18; 631.18; 391.09 | tenuifoliside E | [19] |
| 57 | 35.950 | 923.3825 (2) | C_86_H_128_O_43_ | 1479.68; 851.85 (2) | onjisaponin L ^#^ | [18] |
| 58 | 36.252 | 946.3814 (2) | C_87_H_130_O_45_ | 874.85 (2); 755.80 (2); 699.21 (2) | onjisaponin X | [20] |
| 59 | 36.880 | 880.3579 (2) | C_82_H_122_O_41_ | - | onjisaponin Vg | [20] |
| 60 | 37.165 | 842.3405 (2) | C_80_H_118_O_38_ | 1317.63; 770.82(2) | onjisaponin Ng | [21] |
| 61 | 37.243 | 908.3665 (2) | C_85_H_126_O_42_ | 836.84 (2) | onjisaponin J ^#^ | [18] |
| 62 | 37.470 | 967.3739 (2) | C_90_H_136_O_45_ | 1493.68; 895.85 (2); 865.85 (2); 755.31; 669.21 | onjisaponin Tg | [18] |

^#^: confirmed with authentic compounds


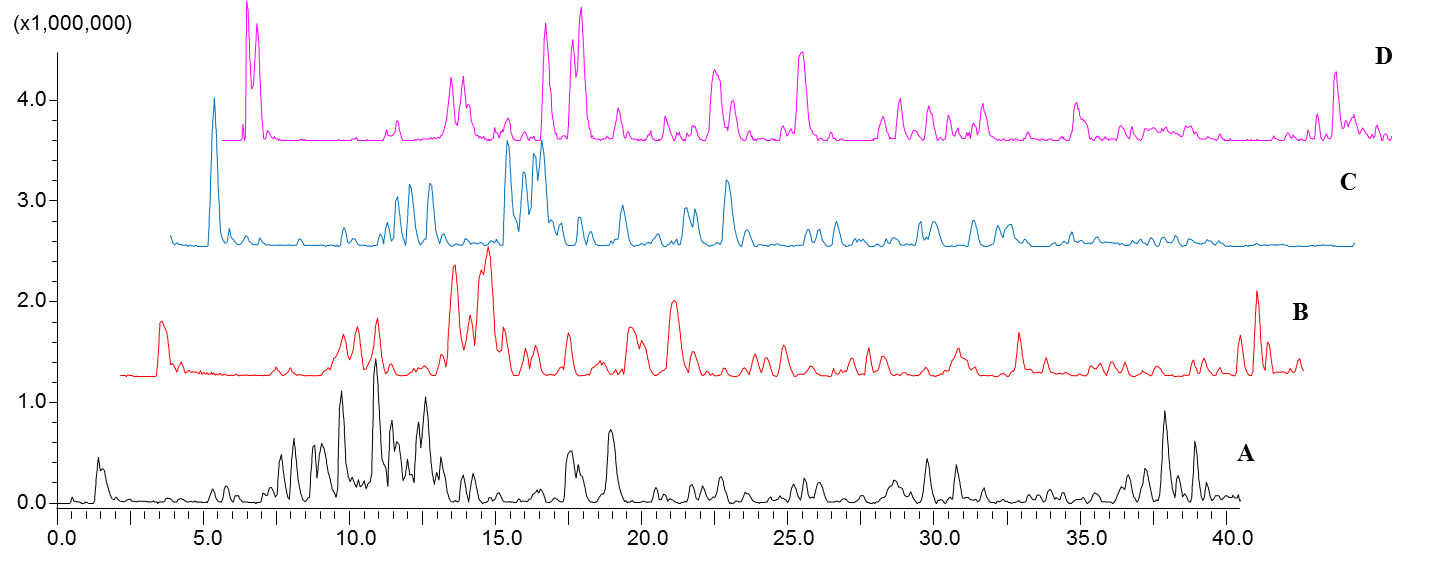


**Fig. S1** Base peak chromatograms of the extracts afforded by PWWE module (A), sonication with 70% aqueous methanol (B), sonication with pure water (C), and reflux with 70% aqueous methanol (D) using LC-IT-TOF-MS.

**
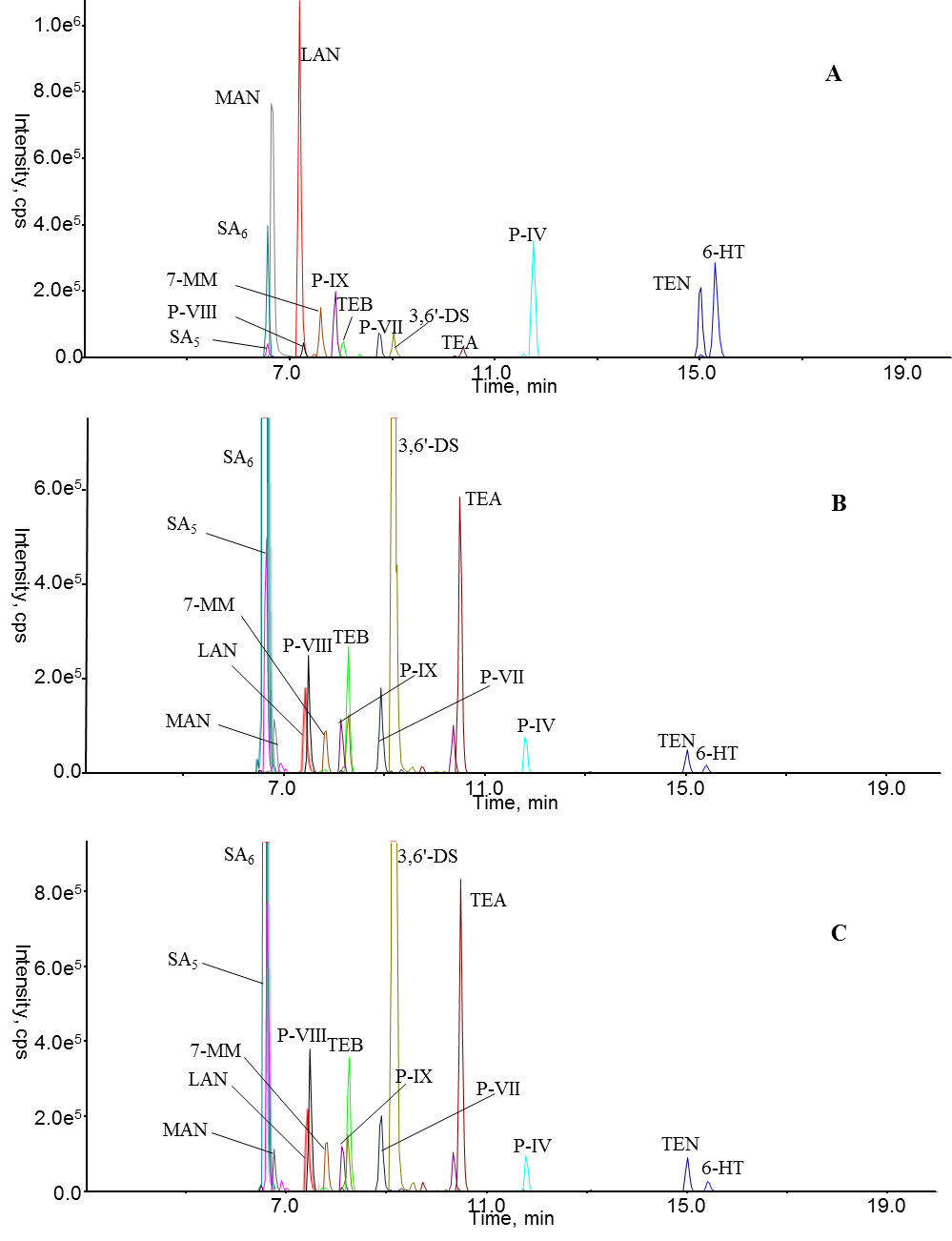
**

**Fig. S2** Representative overlaid extracted ion current (EIC) chromatograms of mixed standard solution (A), selected extract (PR4) afforded by sonication with 70% aqueous methanol (B), and selected extract (PR4) afforded by reflux with 70% aqueous methanol (C) using regular LC-MS/MS. 6-HT, 6-hydroxy-1,2,3,7–tetra methoxyxanthone; LAN, lancerin; MAN, mangiferin; 7-MM, 7-*O*-methoxyl -mangiferin; SA_5_, sibiricose A_5_; SA_6_, sibiricose A_6_; P-IX, polygalaxanthone IX; P-IV, polygalaxanthone IV; P-VIII, polygalaxanthone VIII; P-VII, polygalaxanthone VII; TEB, tenuifoliside B; TEN, tenuifolin; TEA, tenuifoliside A; 3,6′-DS, 3,6′-disinapoyl sucrose.

**
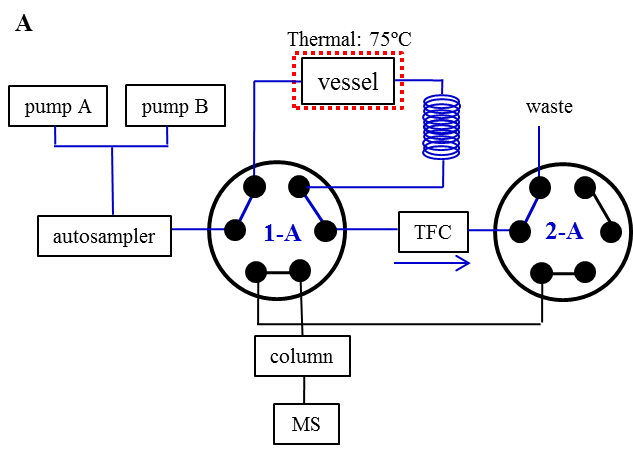
**

**
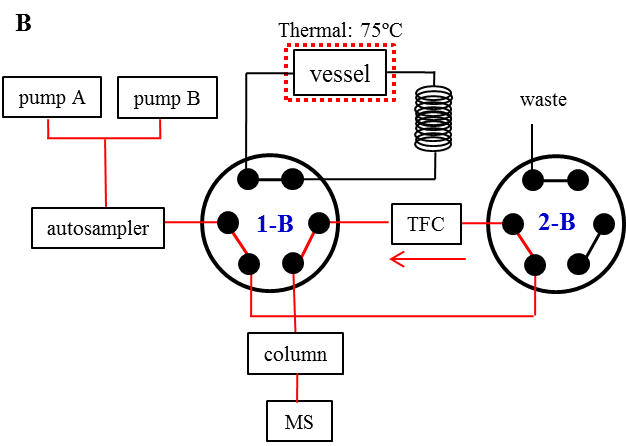
**

**Fig. S3** Brief schematic of modified online PWWE-TFC-LC-MS/MS platform when only two solvent delivery units are available. (A), extraction phase, and the fluid passage was highlighted with blue lines. (B) elution phase, and the fluid passage was highlighted with red lines.

**
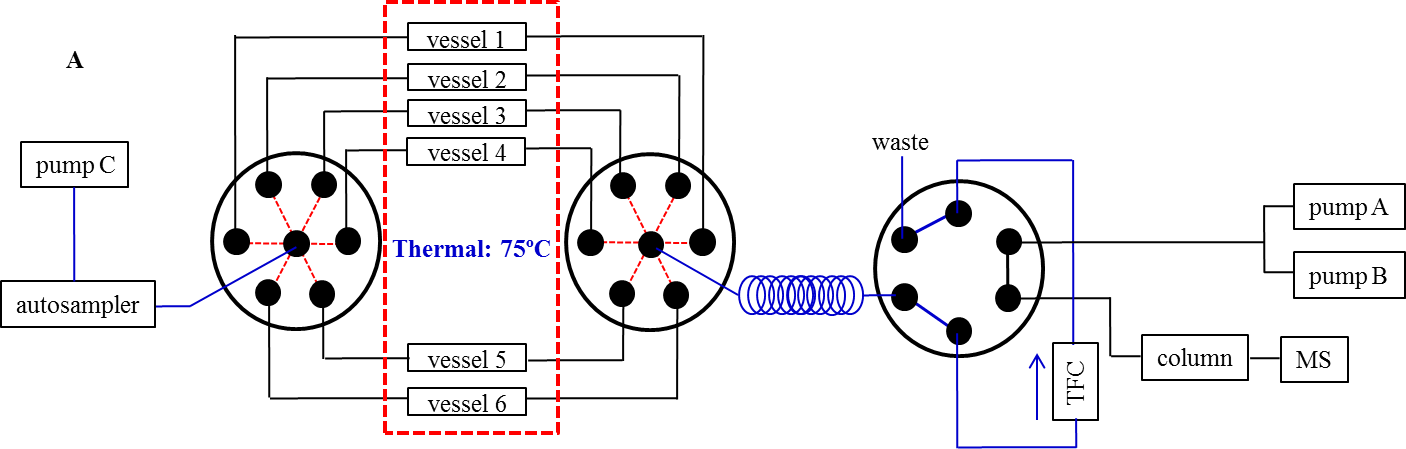
**

**
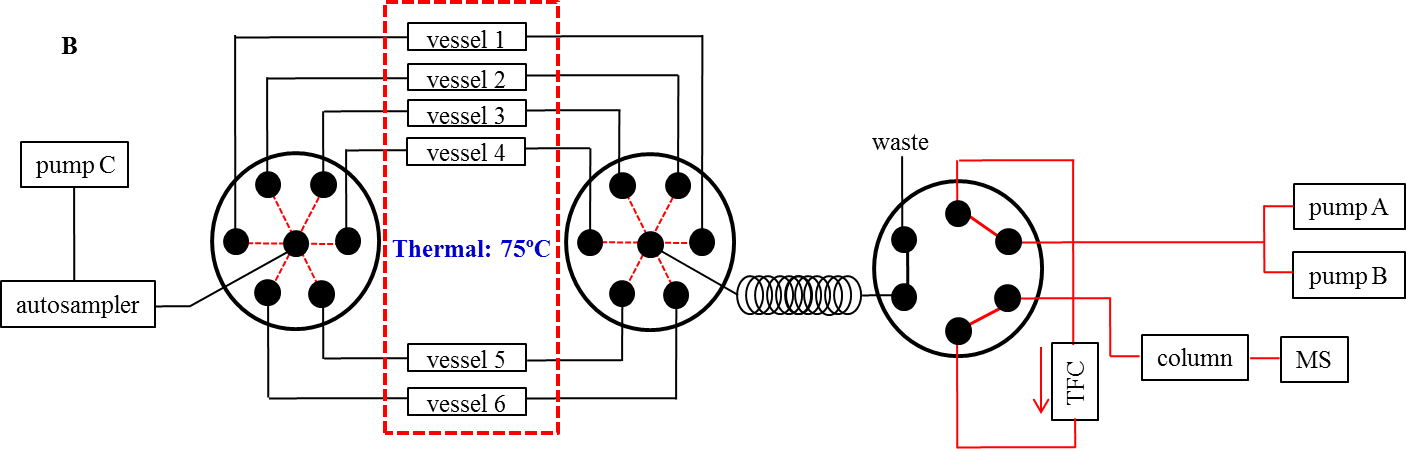
**

**Fig. S4** Brief schematic of modified online PWWE-TFC-LC-MS/MS platform when two 6-channel/6-port electronic valves are available. (A), extraction phase, and the fluid passage was highlighted with blue lines. (B) elution phase, and the fluid passage was indicated with red lines.

**References**

1. T. Miyase, H. Noguchi and X.M. Chen, *J. Nat. Prod.*, 1999, **62**, 993-996.

2. W. Li, C. Chan, H. Leung, H. Yeung and P. Xiao, *Pharm. Pharmacol. Commun.*, 1998, **4**, 415-417.

3. Y. Jiang, W. Zhang, P. Tu and X. Xu, *J. Nat. Prod.*, 2005, **68**, 875-879.

4. S. Wada, Y. Shimizu, N. Tanaka, R.C. Cambie and J. E. Braggins, *Chem. Pharm. Bull.*, 1995, **43**, 461-465.

5. Y. Ikeya, K. Sugandama, M. Okada and H. Mitsuhashi, *Chem. Pharm. Bull.*, 1991, **39**, 2600-2605.

6. Y.H. Zhou, Y. Jiang, H.M. Shi, Y.P. Chen and P.F. Tu, *Helv. Chim. Acta*, 2008, **91**, 897-903.

7. Y. Jiang and P.F. Tu, *Phytochemistry*, 2002, **60**, 813-816.

8. H. Saiton, T. Miyase and A. Ueno, *Chem. Pharm. Bull.*, 1994, **42**, 1879-1885.

9. T. Miyase and A. Ueno, *Jpn. J. Pharmacogn*., 1993, **47**, 267-278.

10. T. Miyase, Y. Iwata and A. Ueno, *Chem. Pharm. Bull.*, 1991, **39**, 3082-3084.

11. J.C. Li, M. Ono and T. Nohara, *Chem. Pharm. Bull.*, 2000, **48**, 1223-1225.

12. T. Miyase, Y. Iwata and A. Ueno, *Chem. Pharm. Bull.*, 1992, **40**, 2741-2748.

13. D. Zhang, T. Miyase, M. Kuroyanagi, K. Umehara and A. UENO, *Chem. Pharm. Bull.*, 1996, **44**, 810-815.

14. L. Yun, Z. Li, M. Chen, Z. Sun, M. Fan and C. Huang, *J. Pharm. Biomed. Anal.*, 2013, **85**, 1–13.

15. H. Ito, H. Taniguchi, T. Kita, Y. Matsuki, E. Tachikawa and T. Fujita, *Phytochemistry*, 1977, **16**, 1614-1616.

16. S. Pelletier, S. Nakamura and R. Soman, *Tetrahedron*, 1971, **27**, 4417-4427.

17. Y. Ikeya, K. Sugama, M. Okada and H. Mitsuhashi, *Phytochemistry*, 1991, **30**, 2061-2065.

18. J. Liu, X. Yang, J. He, M. Xia, L. Xu and S. Yang, *J. Mass Spectrom.*, 2007, **42**, 861-873.

19. Y. Ikeya, K. Sugama and M. Okada, *Chem. Pharm. Bull.*, 1994, **42**, 2305-2308.

20. C. Li, J. Yang, S. Yu, N. Chen, W. Xue, J. Hu and D. Zhang, *Planta Med.*, 2008, **74**, 133-141.

21. C.J. Li, J.Z. Yang, S.S. Yu., D.M. Zhang, W. Xue, Y.H. Yuan and N.H. Chen, *Chin. J. Nat. Med*., 2011, **9**, 321-328.
